# Photoconversion Optimization of Pulsed-Laser-Deposited p-CZTS/n-Si-Nanowires Heterojunction-Based Photovoltaic Devices

**DOI:** 10.3390/nano10071393

**Published:** 2020-07-17

**Authors:** Zakaria Oulad Elhmaidi, Mohammed Abd-Lefdil, My Ali El Khakani

**Affiliations:** 1 Institut National de la Recherche Scientifique (INRS), Centre-Énergie, Matériaux et Télécommunications, 1650 Blvd. Lionel–Boulet, Varennes, QC J3X-1S2, Canada; zakaria.elhmaidi@emt.inrs.ca; 2 MANAPASE, Physics Department, Faculty of Sciences, Université Mohammed V, P.B.1014 Rabat, Morocco; a-lefdil@fsr.ac.ma

**Keywords:** Silicon nanowires, Cu_2_ZnSnS_4_, CZTS, pulsed laser deposition, heterojunctions, solar cells

## Abstract

We report on the achievement of novel photovoltaic devices based on the pulsed laser deposition (PLD) of p-type Cu_2_ZnSnS_4_ (CZTS) layers onto n-type silicon nanowires (SiNWs). To optimize the photoconversion efficiency of these p-CZTS/n-SiNWs heterojunction devices, both the thickness of the CZTS films and the length of the SiNWs were independently varied in the (0.3–1.0 µm) and (1–6 µm) ranges, respectively. The kësterite CZTS films were directly deposited onto the SiNWs/Si substrates by means of a one-step PLD approach at a substrate temperature of 300 °C and without resorting to any post-sulfurization process. The systematic assessment of the PV performance of the ITO/p-CZTS/n-SiNWs/Al solar cells, as a function of both SiNWs’ length and CZTS film thickness, has led to the identification of the optimal device characteristics. Indeed, an unprecedented power conversion efficiency (PCE) as high as ~5.5%, a V_OC_ of 400 mV, a J_SC_ of 26.3 mA/cm^2^ and a FF of 51.8% were delivered by the devices formed by SiNWs having a length of 2.2 µm along with a CZTS film thickness of 540 nm. This PCE value is higher than the current record efficiency (of 5.2%) reported for pulsed-laser-deposited-CZTS (PLD-CZTS)-based solar cells with the classical SLG/Mo/CZTS/CdS/ZnO/ITO/Ag/MgF_2_ device architecture. The relative ease of depositing high-quality CZTS films by means of PLD (without resorting to any post deposition treatment) along with the gain from an extended CZTS/Si interface offered by the silicon nanowires make the approach developed here very promising for further integration of CZTS with the mature silicon nanostructuring technologies to develop novel optoelectronic devices.

## 1. Introduction

Cu_2_ZnSnS_4_ (CZTS) is a p-type semiconductor that continues to be an attractive material for photovoltaic (PV) devices because of its suitable band gap in the visible (1.6–1.9 eV) [1,2] and its large absorption coefficient (over 10^4^ cm^−1^). The abundance and non-toxicity of the CZTS constituent elements make it a suitable candidate for the replacement of classical PV materials, such as CdTe and CuInGaSe_2_ (CIGS), which suffer from the high cost and scarcity of Ga and In and also the toxicity of Cd. In addition to the PV field, CZTS has also been used for other innovative applications. For instance, Jiang et al. reported the use of CZTS as a photocathode for hydrogen production through water splitting [3]. CZTS was also used by Gour et al. to develop a self-powered photodetector device [4]. Finally, CZTS has been integrated as an effective inorganic hole transport layer (HTL) for perovskite-based solar cells, and showed a comparable behavior to the standard spiro MeOTAD HTL [5,6].

In the classical scheme, CZTS is basically integrated as an absorber layer in the rather complex SLG/Mo/p-CZTS/n-CdS/i-ZnO/ITO/Al multi-layered structure to fabricate solar cells, which have been shown to exhibit interesting PV performance [7,8,9]. However, the fabrication of this CZTS related multilayered architecture requires careful optimization of several laborious processing steps [10,11,12], namely: (**i**) post-sulfurization of the as-deposited CZTS layer, (**ii**) selective chemical treatment of CZTS surface (successive etching by KCN, NH_4_OH and HCl solutions), (**iii**) chemical deposition of the CdS buffer layer followed by soft thermal treatment, (**iv**) CZTS/CdS interface engineering, and (**v**) sputter-deposition of a ZnO/ITO top window contact layer. This rather complex and multi-step processing calls for alternative approaches aimed at exploiting the optoelectronic properties of the p-type CZTS films through their direct association with an appropriate n-type material to form a p-n junction-based PV devices. From this perspective, n-type silicon arises as the natural and most promising candidate to be investigated because of its intrinsic properties and very mature and well-established technology.

So far, there have been few reports on the deposition of CZTS on Si substrates by means of sputtering [13], thermal evaporation [14], or spin coating [15,16] techniques. Following their deposition by these methods, the CZTS films have been sulfurized at high temperatures (≥500 °C) prior to their integration into solar cells. However, the power conversion efficiencies (PCE) of such p-CZTS/n-Si solar cells [13,15,16] remain substantially low (~1%), presumably because of the phase impurity and low crystallinity of CZTS thin films. It is also expected that the sulfurization of the CZTS films at annealing temperatures ≥500 °C can adversely affect the CZTS/Si interface because of the undesirable diffusion of Cu, Zn or Sn into Si, which leads to defect formation in Si [17,18]. To limit such a diffusion and its associated drawbacks, a thin layer of TiN (~25 nm-thick) has been proposed as an effective diffusion barrier to successfully mitigate the in-diffusion of CZTS elements into Si when the post sulfurization is done at 525 °C [19]. Therefore, the growth of high crystalline quality CZTS thin films onto Si substrates, with the desired composition and well-controlled interface at relatively low processing temperatures is highly desirable. In this regard, pulsed laser deposition (PLD) arises as a very promising technique for the growth of CZTS films with the right composition and crystalline structure at relatively low substrate temperatures. Indeed, PLD has been demonstrated to be highly effective for growing highly crystallized PbS nanoparticles at room temperature [20,21]. Moreover, the PLD offers the required process latitude to control the composition, microstructure and hence the optoelectronic properties of the produced thin films by almost independently adjusting the various PLD processing parameters, such as laser fluence, repetition rate, substrate temperature, background gas pressure, and the number of laser ablation pulses [22,23,24]. For the PLD of CZTS films, Vanalakar et al. have specifically underlined the great progress achieved in the PCE of pulsed-laser-deposited CZTS (PLD-CZTS)-based solar cells over the last few years [25]. Indeed, the first PLD-CZTS solar cell was reported by Moriya et al. in 2007 and exhibited a conversion efficiency of 1.7% [26]. Then, Moholkar et al. proposed a parameter optimization strategy that allowed them to increase significantly the PCE to 3.1% in 2011 [27], and to 4.1% in 2012 [28]. In 2017, Cazzaniga et al. reported a PCE of 5.2% [29], which is currently the highest PCE reported for PLD-CZTS solar cells; it is to be recalled here that even if the CZTS films were deposited by means of PLD, all those solar cells were fabricated according to the classical SLG/Mo/p-CZTS/n-CdS/i-ZnO/ITO/Al configuration. The impressive increase (tripling in a decade) in the PCE of CZTS-based solar cells clearly demonstrates the potential of the PLD approach for CZTS film growth. This motivated us to focus on the study and optimization of CZTS film growth on silicon substrates by means of PLD for p-CZTS/n-Si solar cells application. In a previous work [30], we identified 300 °C as a relatively low substrate temperature for growing single-phase kësterite CZTS on Si. Then, we integrated those PLD-CZTS films into p-CZTS/n-Si heterojunction solar cells and evaluated for the first time their PCE, which was found to be ~1.1% [31]. In a second step, the flexibility of the PLD approach enabled us to tune the Zn content of the PLD-CZTS films in situ by concomitantly ablating a stoichiometric CZTS target onto the surface of which a desired number of Zn-strips were affixed [32]. This original approach was shown to be effective in depositing PLD-CZTS films, at the optimal temperature of 300 °C, with the desired metal ratios of $\frac{\left[Cu\right]}{\;\left(\left[Zn\right]+\left[Sn\right]\right)}=0.85$ and $\frac{\left[Zn\right]}{\left[Sn\right]}$ = 1.16, by adding three Zn-strips on the CZTS target. The integration of these films into p-CZTS/n-Si solar cells have doubled the PCE to a value of 2.2%.

All the CZTS/Si-based solar cells developed so far have been fabricated with silicon substrates with flat and smooth surfaces. We report here, for the first time, on the development of novel p-CZTS/n-Si heterojunction solar cells where the n-Si substrate consists of silicon nanowires (SiNWs). Replacing flat silicon by a nanostructured silicon (namely SiNWs) aims at enhancing the photoconversion properties of the p-CZTS/n-SiNWs PV devices through the hugely increased extent of the p-n junction between CZTS and SiNWs, as a result of the nanostructuration of Si. In addition, vertically aligned SiNWs are known to not only enhance the light harvesting efficiency of Si [33,34], but also to ensure a better charge transport by shortening their diffusion length [33,35]. 

By conducting systematic studies in which both the n-SiNWs’ length and the PLD-CZTS film thickness were varied, we were able to identify the optimal experimental conditions leading to achieving efficient PLD-CZTS/n-SiNWs heterojunction solar cells exhibiting a PCE value as high as 5.5%. This PCE value is the highest ever reported for solar cells consisting of PLD-CZTS films in conjunction with n-silicon with a relatively simple processing.

## 2. Materials and Methods

The formation of SiNWs was achieved by means of a two-step metal-assisted chemical etching (MACE) of n-type (100) oriented silicon wafers (400 μm, 1–5 Ω/cm). Firstly, a ~25 nm-thick layer of silver was thermally evaporated onto the cleaned n-Si substrates to serve as a catalyst for the MACE. Subsequently, the Ag-coated Si wafers were immersed, at room temperature, in a chemical etching bath containing H_2_O, HF (48%) and H_2_O_2_ (10%) with a volume ratio of (7:2:1). Different immersion durations were used in order to vary the SiNWs’ length (*L_SiNWs_*). The SiNWs were then rinsed in deionized water, dried gently with N_2_ and immediately fixed on the substrate holder-heater, which was placed parallel to the CZTS target, at a distance of 6 cm, inside the PLD chamber. The PLD vacuum chamber was initially pumped to 10^−2^ Torr with a dry-pump and then turbo-pumped to a working pressure of ~5 × 10^−6^ Torr. On the same substrate holder, n-Si flat substrates and quartz (1” × 1”) slides along with the SiNWs/n-Si substrates were heated in situ at a substrate temperature (T_sub_) of 300 °C and then coated concomitantly by a CZTS film. To deposit the CZTS films, a KrF excimer laser (λ = 248 nm, pulse duration = 15 ns, on-target intensity of ~2 × 10^8^ W/cm^2^, repetition rate = 20 Hz) was focused at an incident angle of 45° onto a rotating 5 cm- diameter CZTS target surface onto which three Zn strips were affixed (corresponding to a Zn to CZTS surface ratio [R*_Zn/CZTS_*] of 24%). The number of Zn strips to be added to the CZTS target in order to achieve the desired CZTS film composition was optimized in a previous study [32]. The thickness of the CZTS films was varied by increasing the number of laser pulses (N*_LP_*) from 10,000 to 30,000 pulses. Prior to each deposition, the target surface was cleaned in situ by ablating its surface for 3 min while appropriately shielding the substrate holder from the laser ablation plume by a shutter. Finally, to fabricate the PV devices, a 150-nm thick ITO and 200 nm-thick Al layers were deposited by sputtering on CZTS and silicon, as front and back electrodes, respectively.

The crystalline structure and chemical bonding of the PLD-CZTS films were investigated by grazing angle X-ray diffraction (using an X’Pert ProX-ray diffractometer at a grazing incidence angle of 1.5°, and employing a Cu Kα (λ = 1.5418 Å) radiation) and Raman spectroscopy (using a Renishaw-inVia spectrophotometer with two different excitation wavelengths of 532 and 785 nm, and at a constant laser power of 2.5 mW). The SiNWs’ length, the thickness and morphology of the CZTS films were characterized by using a scanning electron microscope equipped with an integrated energy dispersive spectroscopy system (SEM-EDS Tescan Vega3 LMH). The same SEM was used in the EDS mode to determine the chemical composition of the CZTS films. The reflectance spectra of the SiNWs coated with CZTS as well as the transmittance spectra of CZTS films (deposited on quartz) were acquired by means of a UV-vis-NIR PerkinElmer Lambda-1050-spectrophotometer equipped with an integrating sphere. Electrical resistivity, carrier concentration, and mobility of the pulsed laser deposited CZTS films were measured at room temperature by an ECOPIA Hall effect system (HMS 5500). Finally, the PV performance of the PLD-CZTS solar cells was assessed by systematically measuring their current density–voltage (J-V) characteristics with an Agilent B2901A unit, under a simulated sunlight (AM1.5) at an intensity of 100 mW/cm^2^.

## 3. Results and Discussion

**Figure 1:**
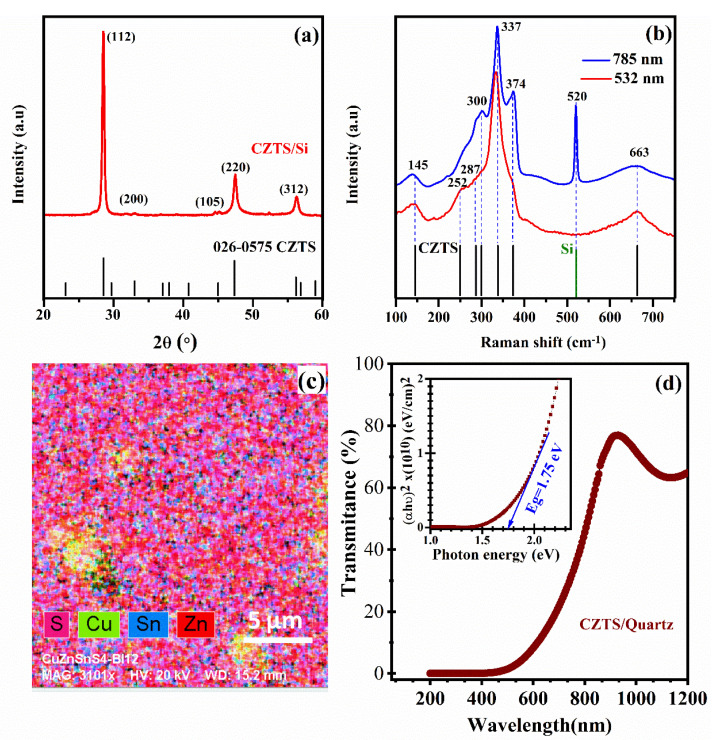
(**a**) Typical XRD pattern of a PLD-CZTS film deposited at N*_LP_* = 15000 onto Si (100) substrates (the underlying tick marks indicate the XRD peak positions of the kësterite crystalline structure according to the JCPDS (26-0575) file); (**b**) Raman spectra of the same PLD-CZTS film at two excitation wavelengths (532 and 785 nm); (**c**) EDX mapping of the S, Cu, Sn and Zn elements forming the PLD-CZTS film; (**d**) UV-Vis Transmittance spectrum of the same PLD-CZTS film deposited on the optically transparent quartz substrate (the inset shows the corresponding Tauc plot from which a bandgap of 1.75 eV is deduced).

In addition to their deposition onto n-SiNWs, the CZTS films were concomitantly deposited onto flat Si and quartz substrates in order to investigate their structural and optoelectronic properties. Figure 1a shows a typical X-ray diffraction (XRD) spectrum of our PLD-CZTS films deposited on flat silicon (100) substrates, at N_LP_ = 15.000, which corresponds to a film thickness of ~490 nm. The observed XRD peaks are characteristic of the kësterite structure (JCPDS No. 026-0575). The very narrow and intense XRD peaks indicate the high crystallinity of the PLD-CZTS films, which seem to exhibit a preferred (112) orientation (with an average crystallite size of ~25 nm, as derived from the Scherrer formula [36]).

Since the XRD peaks positions of the kësterite phase can coincide with those of ZnS and CTS secondary phases, Raman spectroscopy is very convenient for unveiling the existence of such phases if they are really present in the sample. Thus, Raman spectroscopy measurements were performed on the PLD-CZTS films at two excitation wavelengths (532 and 785 nm). All the Raman peaks identified in Figure 1b are due to kësterite CZTS [37,38], and no peak associated with ZnS or CTS (particularly at the 785 nm Raman excitation line) is present. Thus, the XRD and Raman results confirm that our PLD-CZTS films consist of highly crystalline single-phase kësterite CZTS, which is shown here to be successfully grown by a one-step PLD deposition process without resorting to any annealing and/or sulfurization post-deposition treatment. Figure 1c shows the SEM-EDX elemental maps, which confirm a fairly homogenous distribution of the Cu, Zn, Sn and S elements in the PLD-CZTS film. The EDX derived atomic contents of Cu, Zn, Sn and S were found to be 24.2, 16.4, 11.7 and 47.7 at.%, respectively. Consequently, the metal content ratios of $\frac{\left[Cu\right]}{\left(\left[Zn\right]+\left[Sn\right]\right)}$ and $\frac{\left[Zn\right]}{\left[Sn\right]}$ were of ~0.86 and 1.40, respectively, indicating a Cu-poor and Zn-rich film composition, which is the desired one for high-efficiency CZTS PV devices [39]. It is important to note, here, that we have purposely added Zn-strips onto the CZTS target surface in order to achieve the targeted Zn content of the PLD-CZTS films [32]. The UV-Vis transmittance spectrum of the 490 nm thick PLD-CZTS films is shown in Figure 1d. The corresponding optical-absorption coefficient (α) is estimated to be of ~0.8 × 10^5^ cm^−1^ around the 500 nm light wavelength. The optical bandgap (Eg) of these PLD-CZTS films was derived from the standard Tauc plot [${\left(\alpha h\nu \right)}^{2}$ vs hν], and found to be of ~1.75 eV (as displayed in the inset of Figure 1d). Finally, the electrical properties of these PLD-CZTS films were examined by Hall effect measurements and their electrical resistivity, hole concentration and mobility were found to be of 7.0 Ω.cm, 7.5 × 10^16^ cm^− 3^ and 11.8 cm^2^/V.s, respectively. Therefore, our PLD-CZTS films have appropriate structural and optoelectronic properties for PV application [39].

**Figure 2:**
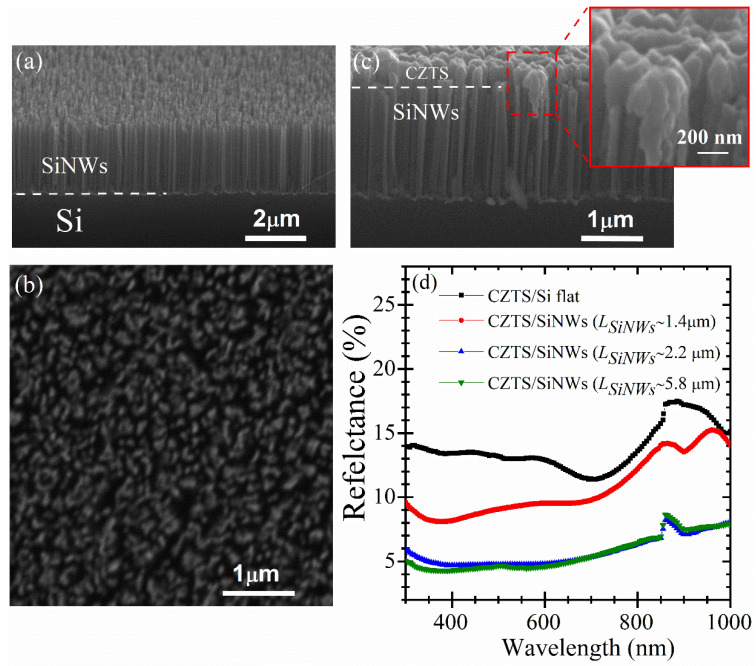
(**a**,**b**) Top and cross-section SEM images of vertically aligned SiNWs formed after 180 s of MACE on n-Si(100) wafer; (**c**) Cross-sectional SEM view of the CZTS/SiNWs heterostructure (the inset image shows a zoomed area of the CZTS/SiNWs interface); (**d**) The reflectance spectra of CZTS films deposited either on flat-Si or on SiNWs with different lengths (*L_SiNWs_* = 1.4, 2.2 and 5.8 µm).

To investigate, first, the effect of the SiNWs’ length on the PV performance of the p-CZTS/n-SiNWs heterojunctions, we arbitrarily fixed the thickness of the CZTS films to ~490 nm. Thus, the length of the SiNWs (*L_SiNWs_*) was varied by adjusting the etching time during the MACE process. For MACE etching times of 120, 180, 270 and 480 s, the average lengths of the SiNWs were of ~1.4, 2.2, 3.3 and 5.8 µm, respectively, while their diameter typically lies in the (60 ± 5) to (25 ± 3) nm range (the longer is the etching time, the thinner is the SiNW diameter and the more porous is the SiNWs array). To assess, as accurately as possible, the effect of etching time on the SiNWs average length, several dozen SEM images were taken from four different etching sets (for a given etching time). The average length for each etching time was found to vary slightly from one sample/set to another, reflecting thus a certain variability in the MACE process. Nevertheless, we found that the statistical variability in the SiNW length lies within an average uncertainty of ± 6%. Typical cross-sectional and top SEM views of the as-produced SiNWs are presented in Figure 2a,b, respectively, for a MACE etching time of 180 s. The SiNWs are seen to form homogeneously onto the Si wafer, and to well-align vertically, with a regular length of (2.20 ± 0.15) µm. It is worth noting that very few samples (~5% of the total) were found to have the SiNWs stripped away or scratched in places (those samples were eliminated, and only those having their full surface uniformly covered with the nanowires were used to deposit the CZTS films).

To form the p-CZTS/n-SiNWs heterojunction, the PLD-CZTS film was deposited on the top of the SiNWs layer, as shown in Figure 2c. A zoomed image of the CZTS/SiNWs interface (inset of Figure 2c) clearly shows that the SiNWs are conformally coated by the CZTS film on their tops and on their side facets (up to a certain depth which remains well within the upper half of the SiNWs’ length). This inter-penetration between CZTS and SiNWs is interesting, as it increases the interfacial extent of the p-n junction and also shortens the pathway for photocharge collection. Figure 2d compares the reflectance spectra of the PLD-CZTS films deposited onto both flat silicon and SiNWs with different *L_SiNWs_*. First of all, it is clearly seen that the nanostructuration of the underlying Si surface (transforming the flat-Si into SiNWs) leads to a significant decrease of the reflectance of the CZTS films over all the investigated (300–1000 nm) spectral range. Secondly, when *L_SiNWs_* is increased, the reflectance diminishes and reaches its minimum for *L_SiNWs_* ≥ 2.2 µm. For example, the reflectance (at 550 nm) of the CZTS-coated-SiNWs samples decreases from ~14% to ~5% when *L_SiNWs_* is increased from 0 (flat-Si) to 2.2 µm. This significant reduction of the reflectance is primarily related to the nanostructured morphology of the underlying SiNWs that enhances light trapping [33,40]. In fact, the antireflection property of SiNWs has been explained by the fact that when the nanowire diameters (in the 25–60 nm range as in the present case) are smaller than the light wavelength, they create a sort of subwavelength structure which behaves like an antireflecting surface [41]. Moreover, Striemera et al. [42] reported that tapered nanowires/nanocones can enhance optical absorption, as they can be considered as an effective medium with a gradual change in refractive index that accompanies the gradual change in SiNWs diameter from the top to their bottom.

**Figure 3:**
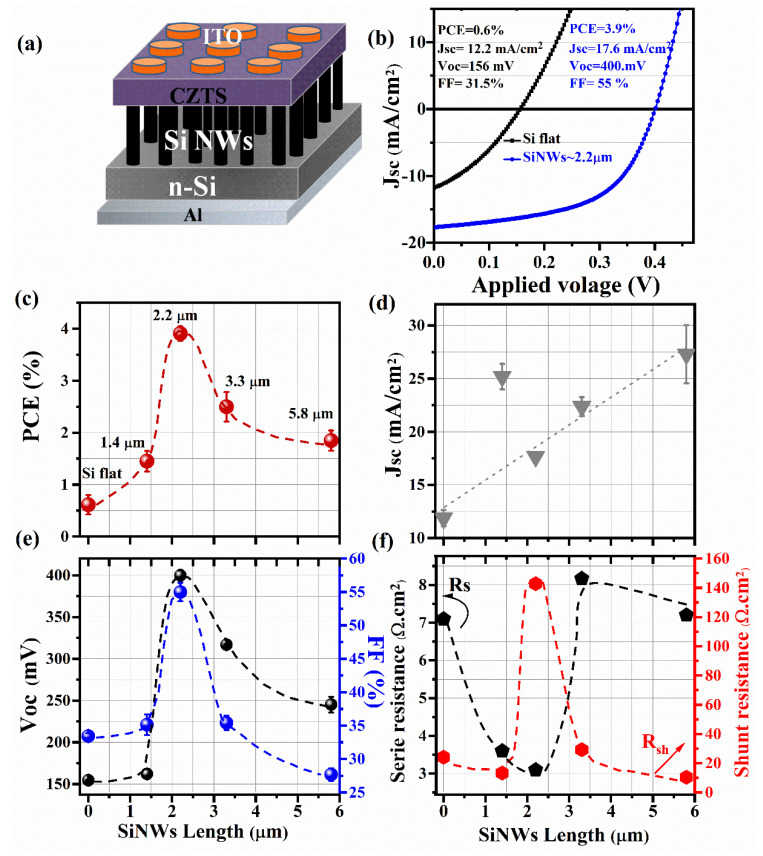
(**a**) Schematic architecture of the ITO/p-CZTS/n-SiNWs/Al solar cell. (**b**) Typical J-V characteristics of the fabricated devices (the blue curve is for CZTS/SiNWs devices and the black one is for CZTS/flat-Si ones). (**c**–**f**) Length dependence of SiNW on the average photovoltaic performances (PCE, J_SC_, V_OC_, FF, and R_S_, R_Sh_,) of the CZTS/SiNWs devices. The error bars represent measurement fluctuations from different devices.

To fabricate the solar cells, a 200 nm-thick Al layer (back-contact) and 150 nm-thick ITO discs (front transparent contact) were sputter-deposited on the back of n-silicon substrates and on the top of the p-CZTS, respectively, as depicted in Figure 3a, which illustrates the structure of our PV devices. This p-CZTS/n-SiNWs heterojunction architecture provides the advantage of being relatively straightforward and does not require complex processing, as in the case of the classical SLG/Mo/p-CZTS/n-CdS/i-ZnO/ITO/Al multi-layered structure. Figure 3b shows the current density–voltage (J-V) characteristics, measured under simulated sunlight (AM1.5), of our typical p-CZTS/n-SiNWs PV-devices prepared with SiNWs length of ~2.2 µm, and compared to the CZTS/Flat-Si reference device (without any Si nanostructuration). From the J-V curves, it can be clearly seen that the overall PV performance of the device has been significantly enhanced after the nanaostructuration of Si. Indeed, the flat Si-based devices show a poor PCE of 0.60%, while the CZTS/n-SiNWs ones (with *L_SiNWs_* of ~2.2 µm) exhibit a significantly higher PCE of 3.9%. Figure 3c–f summarizes the effects of the SiNWs lengths on the solar cell parameters, namely power conversion efficiency (PCE), open circuit voltage (V_OC_), short circuit current density (J_SC_), fill factor (FF), series resistance (R_S_) and shunt resistance (R_Sh_) of the fabricated devices.

As a general trend, the J_SC_ is seen to increase monotonically with increasing *L_SiNWs_* (Figure 3c), this could be attributed to the increasing light trapping and its subsequent absorption as the length of the SiNWs increases, which would lead to more photogenerated carriers and ultimately to a higher produced photocurrent. Astonishingly, the J_SC_ at *L_SiNWs_* = 1.4 µm is found to be as high as that measured at *L_SiNWs_* = 5.8 µm. Even if we do not have a clear-cut interpretation for such a behavior, we cannot exclude the possibility of some leakage current that can contribute to increase the measured photocurrent. This is consistent with the fact that the overall PCE of the devices (at *L_SiNWs_* = 1.4 µm) remains relatively low even if their associated J_SC_ is unexpectedly high. In fact, Figure 3c summarizes the PCE variation of the CZTS/n-SiNWs-based devices as a function of *L_SiNWs_*. It is clearly seen that the PCE of the CZTS/n-SiNWs devices increases as the SiNWs’ length is increased and reaches a maximum of 3.9% for *L_SiNWs_* = 2.2 µm and then decreases for longer SiNWs (*L_SiNWs_* ≥ 3.3 µm). This length dependence of the SiNWs on the PCE is consistent with that of both V_OC_ and FF (Figure 3e). Indeed, the maximum values of both V_OC_ and FF (of 400 mV and 55%, respectively) were also obtained for *L_SiNWs_* = 2.2 µm. The significant decrease of the FF from 55% to 27% when *L_SiNWs_* was increased from 2.2 to 5.8 µm can be particularly assigned to the series resistance (R_S_) of the devices, which is found to increase from ~3 to 7 Ω.cm^2^. Inversely, when *L_SiNWs_* is increased from 2.2 to 5.8 µm, the shunt resistance of the devices decreases from ~143 to 10 Ω.cm^2^. All these above-discussed results show that the photo-electrical behavior of the CZTS/n-SiNWs PV devices is significantly affected by the length of the SiNWs. More interestingly, the present study enabled us to point out the existence of an optimum length, namely *L_SiNWs_* = 2.2 µm, that yields the maximum PCE of 3.9%. The existence of such an optimal SiNWs length can be interpreted as the best trade-off between the highest light trapping and absorption (which is favored by longer SiNWs) and the most efficient collection of the generated photocharges (which is more secured by shorter SiNWs). As a matter of fact, it has been demonstrated that the minority carrier lifetime in MACE produced SiNWs significantly decreases as the length of SiNWs is increased (for instance, it decreases from 62 to 38 µs when *L_SiNWs_* is increased from ~2 to ~6 µm) [43].

**Figure 4:**
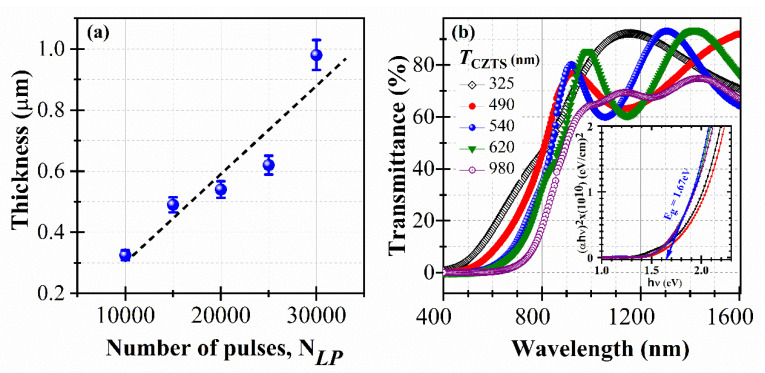
(**a**) Variation of thickness of the PLD-CZTS films as a function of the used N_LP_; (**b**) Optical transmittance spectra of the PLD-CZTS films with their associated Tauc plots (inset) for the various film thicknesses ranging from 325 to 980 nm.

In an effort to further increase the PCE of our p-CZTS/n-SiNWs devices, we fixed the SiNWs length at the above-determined optimal value of *L_SiNWs_* ~2.2 µm, and varied the CZTS film thickness (*T_CZTS_*). Thus, CZTS films with *T_CZTS_* values of 325, 490, 540, 620 and 980 nm were deposited simultaneously onto n-SiNWs (*L_SiNWs_* ~2.2 µm) and quartz substrates by varying the number of laser pulses (N*_LP_*) from 10,000 to 30,000 pulses. The thickness of the CZTS films was systematically measured on several cross-sectional SEM images of each sample. Figure 4a depicts the thickness variation of the CZTS films as a function of N*_LP_*. The CZTS film thickness is seen to increase almost linearly with N*_LP_* (or deposition time) with an average deposition rate of 0.032 ± 0.004 nm/laser pulse. This is rather consistent with the pulsed nature of the laser ablation process, which tends to grow the films in a cumulative way pulse after pulse. Figure 4b shows the optical transmittance spectra of CZTS films deposited onto quartz substrates for the different thicknesses. It is clearly seen that the onset of absorption of the CZTS redshifts to higher wavelengths as *T_CZTS_* is increased from 325 to 980 nm. Their corresponding Tauc plots (inset of Figure 4b) consistently confirms the decrease of the bandgap of the PLD-CZTS films (from 1.8 to 1.6 eV) as their thickness is increased (from 325 to 980 nm, respectively). Such a bandgap decrease with film thickness has already been reported for chemically synthesized CZTS films [44]. In fact, the density of structural defects in CZTS films tends to increase as they get thicker, which would populate the gap with localized states near the valence band. Those localized states may merge with the band edge, resulting in the observed narrowing of the bandgap [45].

**Figure 5:**
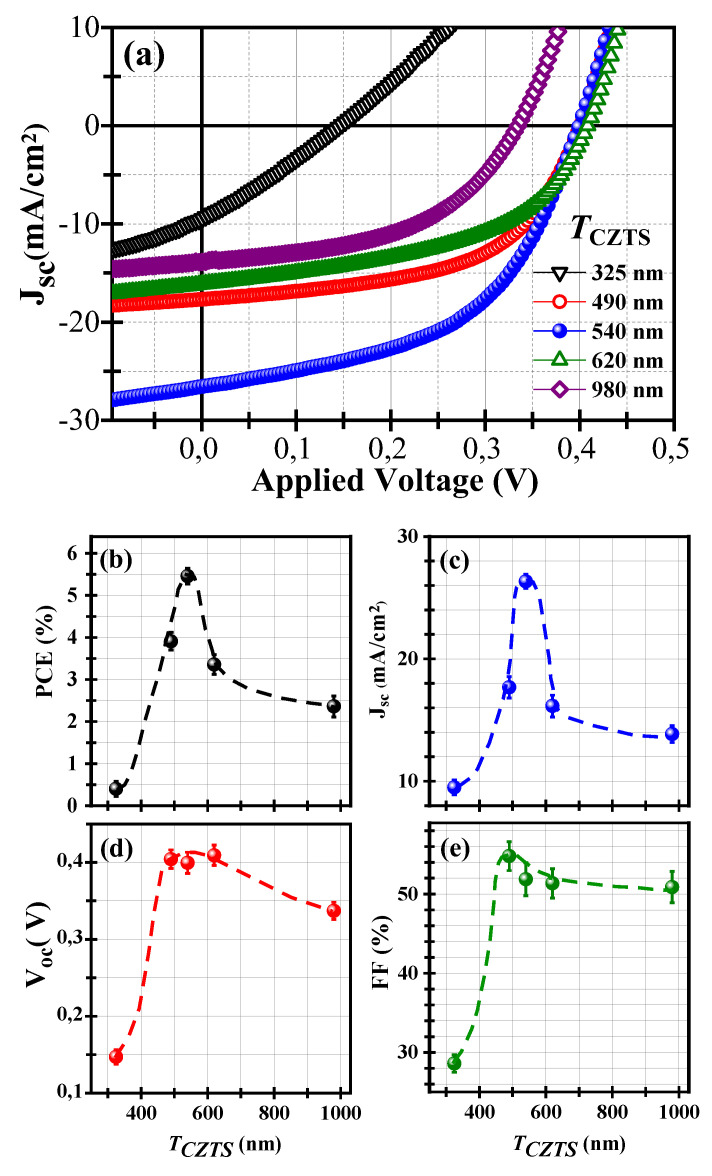
(**a**) J-V characteristics of the CZTS/SiNWs PV-devices as a function of the CZTS film thickness (*T_CZTS_*); (**b**–**e**) *T_CZTS_* dependence of the PV parameters (PCE, J_SC_, V_OC_ and FF) of the CZTS/SiNWs solar cells.

CZTS films with different thicknesses were integrated into solar cells (with the same architecture depicted in Figure 3a), and their PV performance was systematically investigated. Figure 5a–e depicts the J-V characteristics of the devices and their associated PV parameters as a function of *T_CZTS_*. It can be clearly seen from the J-V curves that CZTS thickness has a significant effect on the solar cells’ performance. For the thinnest CZTS films (*T_CZTS_* = 325 nm), the devices exhibited poor performance, with a PCE value of ~0.4%. As *T_CZTS_* increased, both J_SC_ and PCE were found to increase until they reached their maximum values (26.3 mA/cm^2^ and 5.5%, respectively) around the CZTS thickness of *T_CZTS_* = 540 nm, and then drop for thicker films down to 13.8 mA/cm^2^ and 2.3%, respectively, for the thickest CZTS films (*T_CZTS_* ~980 nm); see Figure 5b,c. On the other hand, Figure 5d,e exhibits similar *T_CZTS_* dependences, where both V_OC_ and FF are seen to increase abruptly (from 150 to 400 mV and from 29% to 55%, respectively) when *T_CZTS_* is increased from 325 to ~500 nm and then decrease very slowly to reach ~350 mV and ~50%, respectively, for the thickest films (*T_CZTS_* = 980 nm). These results clearly indicate *T_CZTS_* ~540 nm to be the optimal CZTS film thickness, which yields the highest PCE in our CZTS/SiNWs PV devices. The existence of an optimal *T_CZTS_* that maximizes the PCE can be interpreted as the thickness that offers the highest light absorption while not hindering the collection of generated photocharges. For thinner CZTS films, the conformal coating of the SiNWs may not be complete (limiting thereby the extent of the 3D p-n junction between p-CZTS and n-Si) and only a part of the incoming photons can be absorbed and efficiently converted at the junction. For CZTS thick films (≥620 nm), at least two mechanisms can concur to limit the photoconversion efficiency. Firstly, more photons are absorbed in a thick film, but only some of them can reach the space charge zone at the CZTS/Si interface, since the light penetration depth (*L_D_* = 1/α) for the longest absorbable wavelengths is ~420 nm (this value can be calculated by considering an α value of ~0.238 10^5^ cm^−1^ at 730 nm; 730 nm being the longest wavelength that can be absorbed by the PLD-CZTS films having a bandgap of ~1,7 eV). Shorter wavelengths (≤730 nm) are naturally absorbed at smaller *L_D_* values. Secondly, the thicker the films are, the higher the chances are for the generated photocharges to recombine before reaching the collecting electrodes (the minority carrier-diffusion length in CZTS is of ~350 nm [46,47]). Therefore, there is a trade-off between increasing the light absorption (thicker CZTS films with smaller bandgap) and securing photocharge collection (thinner films where the density of structural defects is lesser), which appears to be achieved in our case at the optimal CZTS film thickness of ~540 nm, which yielded the highest PCE of 5.5%. This PCE is the highest value reported so far for PLD-CZTS-based solar cells.

**Figure 6:**
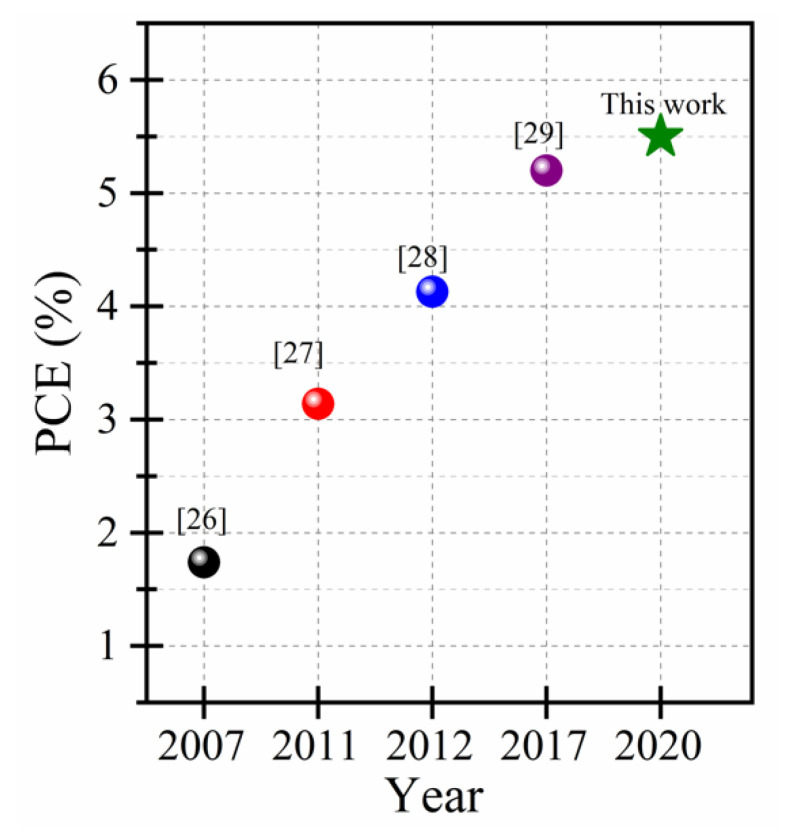
Progress achieved in the power conversion efficiency for PLD-CZTS-based solar cells over the (2007–2020) time period. The green star corresponds to the average PCE achieved in this work.

Indeed, Figure 6 shows the PCE improvement achieved over the last decade by using exclusively pulsed-laser-deposited CZTS films. Indeed, the PCE of PLD-CZTS-based solar cells has been increased from 1.75% in 2007 [26] to 5.5% in the present work. It is worth undescoring that because of the very scarce literature on the subject of PLD-CZTS-based solar cells, the PCE comparison is performed here simply on the basis of where PLD-CZTS films were used as a p-type material to form solar cells and compare their overall PCE regardless of the n-type material or device configuration used. As such, Figure 6 summarizes the state-of-the-art of the PCE of PLD-CZTS films-based solar cells, with all device configurations combined. In fact, except for our present work, all the PLD-CZTS films were integrated into solar cells with the classical multilayer complex structure, that is, SLG/Mo/MoS_2_/p-CZTS/n-CdS/i-ZnO/ITO/Al [26,27,28,29]. Thus, the originality of the present study lies not only in the slight improvement of the PCE (reaching 5.5%), but most importantly in the relative simplicity of fabrication of our CZTS/SiNWs devices, which is based on the direct PLD deposition of a CZTS layer onto SiNWs formed on the standard silicon substrates without resorting to any annealing, post-deposition treatment, and/or complex multilayer device management. This work thereby opens new avenues for the further development of highly efficient PV devices based on the conjunction of PLD-CZTS films with the mature silicon technology.

## 4. Conclusions

We proposed an original concept of p-CZTS/n-SiNWs heterojunction solar cells based on the direct pulsed laser deposition of CZTS films onto vertically aligned silicon nanowires. Compared to the standard multilayered (SLG/Mo/MoS_2_/p-CZTS/n-CdS/i-ZnO/ITO/Al) solar cell architecture, our approach stands out due to its relative ease of device fabrication, while capitalizing on the well-established silicon technology options and yet achieving the highest PCE to date. By separately investigating the effects of both the SiNWs’ length and the CZTS film thickness in the performance of CZTS/SiNWs PV devices, we were able to identify the optimal device characteristics (i.e., *L_SiNWs_* ~2.2 µm and *T_CZTS_* ~540 nm, respectively) that yielded the highest PCE value (~5.5%) ever reported for PLD-CZTS-based solar cells. This optimal SiNWs length and CZTS thickness are thought to correspond to the best conditions that maximize the light absorption in the devices while still ensuring an efficient transport of the generated photocharges to the collecting electrodes with a minimum of recombinations. Finally, in addition to the record PCE achieved here, most important is the demonstration of the potential of combining the mature silicon technology (particularly through the nanostructuration of silicon used here) with the one-step PLD deposition (in contrast to multi-processing steps required in classical multi-layered CZTS solar cells) of CZTS recently developed in our laboratory [32] to achieve high-performance CZTS/SiNWs photovoltaic devices.
